# Relationship between medical students’ career priority and specialty choice: A nationwide multicenter survey

**DOI:** 10.1002/jgf2.349

**Published:** 2020-06-11

**Authors:** Kenya Ie, Akiko Murata, Masao Tahara, Manabu Komiyama, Shuhei Ichikawa, Yousuke C. Takemura, Hirotaka Onishi

**Affiliations:** ^1^ Department of General Internal Medicine Tama Municipal Hospital Kanagawa Japan; ^2^ Department of General Internal Medicine St. Marianna University School of Medicine Kanagawa Japan; ^3^ Family Practice Center of Okayama Okayama Japan; ^4^ Iwakura Station Tahara Clinic Kyoto Japan; ^5^ Thank You All, Family Clinic Hiratsuka Kanagawa Japan; ^6^ Department of Education and Research in Family and Community Medicine Mie University Graduate School of Medicine Mie Japan; ^7^ Department of Family Medicine Mie University School of Medicine Mie Japan; ^8^ Department of Family Medicine Graduate School of Medical and Dental Sciences Tokyo Medical and Dental University Tokyo Japan; ^9^ International Research Center for Medical Education Graduate School of Medicine The University of Tokyo Tokyo Japan

**Keywords:** career choice, career priority, medical students, Surveys and Questionnaires

## Abstract

**Background:**

The shortage of physicians in several specialties has been brought to public attention in several countries. However, little is known about factors affecting medical students’ specialty choice. The objectives of our study were to illustrate medical students’ career priority clusters and to assess their association with specialty preference.

**Methods:**

We conducted a nationwide multicenter survey in 2015 at 17 medical schools. The study participants were asked their top three specialty preferences, demographic characteristics, and 14 career priority questions. Multilevel logistic regression models were used to determine the effect of each variable on student career choice.

**Results:**

A total of 1264 responses were included in the analyses. The top five specialty choices were internal medicine: 833, general practice: 408, pediatrics: 372, surgery: 344, and emergency medicine: 244. An exploratory factor analysis mapped the 14 career priorities into 3‐factor solution: “primary care orientation,” “advanced and specific care,” and “personal life orientation.” Multilevel logistic regression models yielded satisfactory accuracy with the highest ROC curve (AUROC) noted in surgery (0.818), general practice (0.769), and emergency medicine (0.744). The career priorities under “primary care orientation” had positive association with choosing general practice, emergency medicine, internal medicine, and pediatrics. The “advanced and specific care” career priorities facilitated surgery and emergency medicine choice, while reducing the likelihood of choosing less procedure‐oriented specialties, such as internal medicine, general practice, and pediatrics.

**Conclusions:**

Our results demonstrated medical students’ career priorities and their association with specialty preference. Individualized career support may be beneficial for both medical students and each specialty fields.

## Graphical abstract

The top five specialty choices among medical students were internal medicine, general practice, pediatrics, surgery, and emergency medicine. An exploratory factor analysis mapped 14 career priorities into 3‐factor solution: “primary care orientation,” “advanced and specific care,” and “personal life orientation.” The career priorities under “primary care orientation” had positive association with choosing general practice, emergency medicine, internal medicine, and pediatrics, while “advanced and specific care” career priorities facilitated surgery and emergency medicine choice.

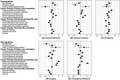

## 1 INTRODUCTION

With rapid aging and changes in disease patterns, Japan has faced a severe shortage of physicians in several specialties. Based on work hours required to fulfill patient needs, the Japanese Ministry of Health, Labour and Welfare began to estimate the number of physicians needed in each specialty, including general practice/family medicine.^1^ Additionally, the Japanese Medical Specialty Board began to certify medical specialists as of 2018 to ensure the quality of postgraduate training and to remedy uneven distribution across specialties.^2^

Several factors have been shown to influence medical students’ specialty choices, including demographic factors (eg, gender,^3^, ^4^, ^5^ birthplace,^3^, ^4^, ^6^ and physician parents^6^, ^7^) and career priorities.^3^, ^8^, ^9^ Moreover, it has been pointed out that medical students may choose from a group of related specialties based on a cluster of socioeconomic and occupational features.^6^, ^9^, ^10^ Thus, it is necessary to clarify the relationship between demographic and occupational features and examine their effect on career preferences. However, little is known about factors affecting medical students’ specialty choices. The objectives of this study therefore were to illustrate medical students’ career priorities and to assess their association with specialty preference.

## 2 METHODS

### 2.1 Study design and sampling

We conducted a cross‐sectional study using data collected for the Japan MEdical Career of Students (JMECS) study, a nationwide observational study conducted from April to December 2015, at 17 medical schools in Japan. The original JMECS study included a total of 1264 medical students who had enrolled in their final year during April 2015. Medical students were informed that their participation was voluntary and that they were deemed to give informed consent for participation upon survey submission. The study protocol was approved by the Institutional Ethical Committee of Mie University Graduate School of Medicine (No. 1482). The protocol details and results of the original JMECS study have been published previously.^7^

### 2.2 Outcome measurement

We used a 21‐item questionnaire that included a question about specialty choice. The primary outcome measured interest in 19 specialty fields as career options: internal medicine, pediatrics, dermatology, psychiatry, surgery, orthopedics, obstetrics and gynecology, ophthalmology, otolaryngology, urology, neurosurgery, radiology, anesthesiology, pathology, clinical laboratory, emergency medicine, plastic surgery, rehabilitation, and general practice. Other variables included in the original JMECS study were students’ demographics (age, gender, birthplace, other academic or professional experiences prior to medical school, physician parent, and plan to inherit an existing practice) and 14 career priority questions with 6‐point Likert scale answers ranging from 1 (strongly disagree) to 6 (strongly agree).

### 2.3 Statistical analysis

The primary outcome variable was dichotomized based on whether or not each specialty field was included in up to three career choices. Explanatory variables excluding age were treated as nominal variables. To investigate the structure of career priorities, an exploratory factor analysis with minimum residual solution and Harris‐Kaiser's orthoblique rotation were conducted. The number of factors was determined using a scree plot, which showed the eigenvalues on the *y*‐axis and the number of factors on the *x*‐axis. Items with factor loading below 0.4 or above 0.4 for two or more domains were eliminated. A series of multilevel logistic regression analyses were conducted to examine the effect of each demographic and career priority variable on student career choice. School ID was set as level 2 and subject ID as level 1. Odds ratios and 95% confidential intervals were calculated from estimates and corresponding standard errors. Each model for five specialties was validated using a 2 × 2 table and AUROC. All the analyses were conducted with R 3.5.3 in RStudio 1.2.1335, and with following packages: haven,^11^ tidyverse,^12^ psych,^13^ GPArotation,^14^ lme4,^15^ pROC,^16^ and tableone.^17^

## 3 RESULTS

### 3.1 Description of respondents

Of the 1264 Japan MEdical Career of Students (JMECS) participants, the top five specialties were found to be internal medicine (833 [65.9%]), general practice (408 [32.3%]), pediatrics (372 [29.4%]), surgery (344 [27.2%]), and emergency medicine (244 [19.3%]). Characteristics of the overall study participants and by top 5 specialty choices are presented in Table 1. Those choosing surgery and emergency medicine included a higher proportion of male students (77.6% and 77.0%, respectively) as compared to internal medicine, general practice, and pediatrics. The proportion of students with a physician parent was lower among those choosing general practice (27.9%) and emergency medicine (27.5%) as compared to the overall participants (32.1%).

**Table 1:** Characteristics of overall participants and top 5 specialty choices

	Total (N = 1264)	Internal medicine (N = 833)	General Practice (N = 408)	Pediatrics (N = 372)	Surgery (N = 344)	Emergency medicine (N = 244
Demographics; No. (%) of students						
Age, median (range), y	24 (23‐58)	24 (23‐58)	24 (23‐58)	24 (23‐52)	24 (23‐43)	24 (23‐45)
Sex (male)	838 (66.3)	538 (64.6)	274 (67.2)	240 (64.5)	267 (77.6)	188 (77.0)
Hometown						
Urban	267 (21.1)	161 (19.3)	72 (17.7)	79 (21.3)	82 (23.8)	62 (25.4)
Relatively urban	287 (22.7)	193 (23.2)	102 (25)	89 (23.9)	64 (18.6)	52 (21.3)
Relatively rural	401 (31.7)	268 (32.2)	130 (31.9)	118 (31.7)	109 (31.7)	67 (27.5)
Rural	309 (24.5)	211 (25.3)	104 (25.5)	86 (23.1)	89 (25.9)	63 (25.8)
Other academic or professional experiences prior to medical school	286 (22.6)	186 (22.3)	102 (25)	92 (24.7)	89 (25.9)	58 (23.8)
Physician parent	406 (32.1)	277 (33.3)	114 (27.9)	112 (30.1)	107 (31.1)	67 (27.5)
Intent to inherit existing practice	143 (11.3)	90 (10.8)	55 (13.5)	37 (9.9)	36 (10.5)	26 (10.7)
Career priorities^a^, mean (SD)						
Mastering advanced procedures	4.83 (1.00)	4.71 (1.00)	4.60 (1.03)	4.66 (1.04)	5.31 (0.81)	5.03 (0.97)
Work‐life balance	4.89 (0.93)	4.92 (0.91)	4.90 (0.89)	4.95 (0.89)	4.65 (1.00)	4.71 (1.01)
Frequent patient communication	4.82 (0.89)	4.85 (0.86)	5.01 (0.82)	4.97 (0.79)	4.83 (0.85)	4.87 (0.91)
Opening own clinic	3.33 (1.35)	3.42 (1.31)	3.48 (1.34)	3.38 (1.27)	2.98 (1.36)	3.11 (1.43)
Involvement in preventive medicine	4.06 (1.13)	4.13 (1.09)	4.40 (1.04)	4.16 (1.12)	3.73 (1.16)	4.14 (1.21)
Involvement in terminal care	3.77 (1.15)	3.86 (1.09)	4.06 (1.03)	3.78 (1.14)	3.54 (1.19)	3.68 (1.21)
Acute care rather than chronic care	4.11 (1.06)	3.98 (1.01)	3.96 (1.02)	4.09 (0.98)	4.58 (0.97)	4.71 (0.99)
Not treat patients with psychosocial problems	2.75 (1.19)	2.71 (1.16)	2.50 (1.14)	2.58 (1.17)	2.78 (1.12)	2.70 (1.19)
Income	4.17 (1.00)	4.14 (0.97)	4.04 (1.08)	4.10 (0.95)	4.10 (1.04)	4.03 (1.08)
Access to advanced medical fields	4.28 (0.98)	4.20 (0.96)	4.06 (0.97)	4.22 (0.92)	4.64 (0.86)	4.36 (1.03)
Clinical diagnostic reasoning	4.31 (1.00)	4.41 (0.93)	4.60 (0.95)	4.32 (0.94)	4.20 (1.07)	4.53 (1.04)
Depth rather than breadth of practice	3.97 (1.02)	3.88 (0.97)	3.64 (0.97)	3.78 (0.98)	4.19 (1.02)	3.92 (1.09)
Involvement in global health	3.37 (1.13)	3.32 (1.10)	3.45 (1.13)	3.40 (1.09)	3.37 (1.12)	3.51 (1.14)
Community‐oriented practice	4.09 (1.05)	4.17 (1.01)	4.47 (0.98)	4.19 (0.97)	3.90 (1.06)	4.18 (1.07)

^a^ “Please select one of the following options which best describes your thoughts regarding your career priorities.” (1 = strongly disagree, 6 = strongly agree)

### 3.2 Student career priorities

From the 14 career priority questions, “work‐life balance” had the highest agreement (mean 4.89 [SD 0.93]) on a 6‐point Likert scale, followed by “mastering advanced procedures” (mean 4.83 [SD 1.00]) and “frequent patient communication” (mean 4.82 [SD 0.89]). Students who chose surgery and emergency medicine gave higher priority to “mastering advanced procedures” and “acute care rather than chronic care,” while those who chose general practice and pediatrics generally gave higher scores to “frequent patient communication.” Exploratory factor analysis revealed three major factors: “primary care orientation,” “advanced and specific care,” and “personal life orientation,” while two items with factor loading below 0.4 were eliminated. Table 2 presents factor loadings of career priorities based on Harris‐Kaiser's orthoblique rotation.

**Table 2:** Final rotated factor loadings for 12 items comprising the scale^a^ (n = 1264)

Domain	Career priorities	1	2	3
“Primary care orientation”	Involvement in preventive medicine	**0.655**	−0.069	0.145
	Community‐oriented practice	**0.634**	−0.135	0.042
	Involvement in terminal care	**0.599**	−0.103	0.091
	Frequent patient communication	**0.583**	0.023	0.004
	Clinical diagnostic reasoning	**0.423**	0.243	−0.026
“Advanced and specific care”	Access to advanced medical fields	0.099	**0.752**	0.002
	Depth rather than breadth of practice	−0.022	**0.587**	−0.02
	Acute care rather than chronic care	0.158	**0.472**	−0.08
	Mastering advanced procedures	0.16	**0.43**	−0.015
“Personal life orientation”	Work‐life balance	−0.145	0.194	**0.736**
	Opening own clinic	0.147	−0.054	**0.457**
	Income	0.18	−0.021	**0.447**

^a^ Exploratory factor analysis with minimum residual solution (Harris‐Kaiser's orthoblique rotation). The bold value represents factor loadings greater than 0.4.

### 3.3 Factors associated with specialty choice

**Figure 1:**
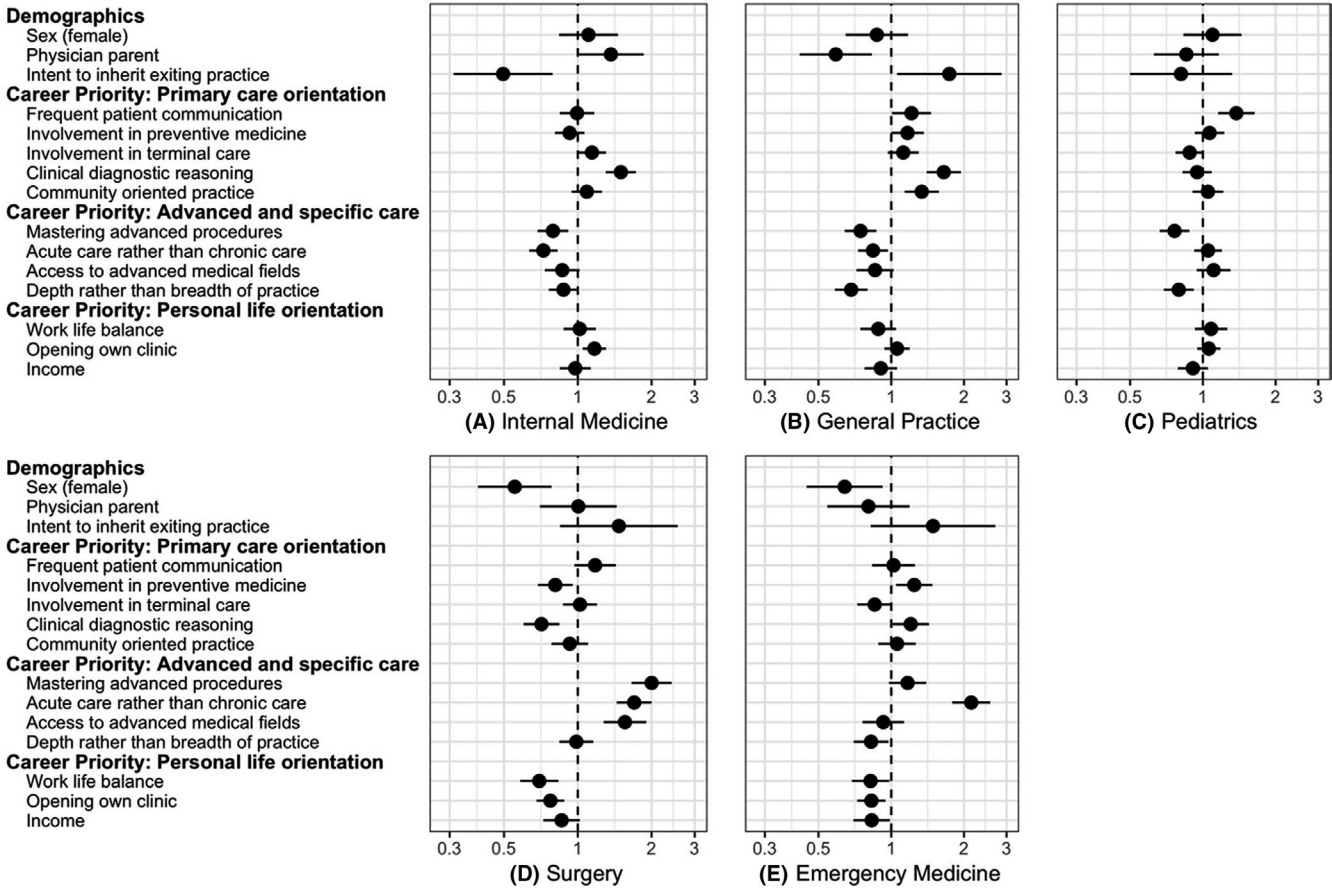
Adjusted odds ratios for specialty preference. The central points of each horizontal line represent the adjusted odds ratios for each item, and the lines demonstrate 95% confidence intervals

Medical students’ demographics and career priorities associated with choosing (a) internal medicine, (b) general practice, (c) pediatrics, (d) surgery, and (e) emergency medicine were elucidated by multilevel logistic regression models using school ID as level 2 and subject ID as level 1 (Table 3; Figure 1). The accuracy of the fitted models ranged from 69.6% (internal medicine model) to 82.4% (emergency medicine model). The highest AUROC was noted in the surgery model, followed by the general practice model and the emergency medicine model.

**Table 3:** Multilevel logistic regression models for specialty choice

	Internal medicine model (N = 833)	General practice model (N = 408)	Pediatrics model (N = 372)	Surgery model (N = 344)	Emergency medicine model (N = 244)
	Adjusted OR (95% CI)	Adjusted OR (95% CI)	Adjusted OR (95% CI)	Adjusted OR (95% CI)	Adjusted OR (95% CI)
Demographics					
Sex (female)	1.11 (0.84‐1.46)	0.87 (0.65‐1.17)	1.10 (0.83‐1.45)	0.55 (0.39‐0.78)^b^	0.64 (0.45‐0.92)^b^
Physician parent	1.37 (1.00‐1.86)^a^	0.59 (0.42‐0.83)^b^	0.85 (0.63‐1.16)	1.00 (0.70‐1.44)	0.81 (0.54‐1.19)
Intent to inherit existing practice	0.50 (0.31‐0.79)^b^	1.74 (1.06‐2.86)^a^	0.81 (0.50‐1.32)	1.47 (0.85‐2.56)	1.49 (0.82‐2.70)
Career priority: “primary care orientation”					
Frequent patient communication	0.99 (0.85‐1.17)	1.21 (1.01‐1.46)^a^	1.38 (1.16‐1.64)^a^	1.18 (0.97‐1.43)	1.02 (0.83‐1.25)
Involvement in preventive medicine	0.93 (0.81‐1.06)	1.17 (1.00‐1.36)	1.07 (0.93‐1.23)	0.81 (0.69‐0.95)^b^	1.25 (1.05‐1.48)^a^
Involvement in terminal care	1.14 (1.00‐1.30)^a^	1.12 (0.97‐1.30)	0.88 (0.77‐1.01)	1.02 (0.87‐1.20)	0.85 (0.72‐1.01)
Clinical diagnostic reasoning	1.50 (1.30‐1.73)^a^	1.65 (1.40‐1.94)^a^	0.95 (0.82‐1.09)	0.71 (0.60‐0.84)^b^	1.20 (1.01‐1.43)^a^
Community‐oriented practice	1.09 (0.94‐1.26)	1.34 (1.14‐1.57)^a^	1.05 (0.91‐1.22)	0.93 (0.78‐1.10)	1.06 (0.88‐1.26)
Career priority: “advanced and specific care”					
Mastering advanced procedures	0.79 (0.69‐0.92)^b^	0.75 (0.64‐0.87)^b^	0.76 (0.66‐0.88)^b^	2.00 (1.66‐2.41)^a^	1.17 (0.98‐1.40)
Acute care rather than chronic care	0.72 (0.63‐0.83)^b^	0.84 (0.73‐0.97)^b^	1.05 (0.92‐1.20)	1.70 (1.44‐2.00)^a^	2.14 (1.79‐2.57)^a^
Access to advanced medical fields	0.86 (0.73‐1.02)	0.86 (0.72‐1.02)	1.11 (0.94‐1.30)	1.56 (1.27‐1.90)^a^	0.93 (0.76‐1.13)
Depth rather than breadth of practice	0.87 (0.76‐1.00)	0.68 (0.59‐0.80)^b^	0.80 (0.69‐0.92)^b^	0.99 (0.84‐1.16)	0.83 (0.70‐0.97)^b^
Career priority: “personal life orientation”					
Work‐life balance	1.02 (0.88‐1.18)	0.88 (0.75‐1.05)	1.08 (0.93‐1.26)	0.70 (0.58‐0.83)^b^	0.82 (0.69‐0.98)^b^
Opening own clinic	1.17 (1.05‐1.30)^a^	1.06 (0.94‐1.19)	1.06 (0.95‐1.18)	0.77 (0.68‐0.88)^b^	0.83 (0.72‐0.95)^b^
Income	0.98 (0.85‐1.13)	0.91 (0.78‐1.06)	0.91 (0.79‐1.05)	0.86 (0.72‐1.02)	0.83 (0.70‐0.99)^b^
AIC	1524.853	1373.734	1510.355	1185.012	1112.107
Sensitivity (%)	91.5	39.7	5.1	42.7	16.4
Specificity (%)	27.4	91.6	97.5	92.0	98.0
Positive predictive value (%)	70.9	69.2	46.3	66.5	66.7
Accuracy (%)	69.6	74.8	70.3	78.6	82.3
AUROC	0.697	0.769	0.641	0.818	0.744

^a^ Significantly increasing the likelihood of choosing the specialty.

^b^ Significantly decreasing the likelihood of choosing the specialty.

* Cutoff = *P*(.5).

#### 3.3.1 Internal medicine

Factors such as having a physician parent (OR 1.37 [1.00‐1.86]) and plans to open one's own clinic (OR 1.17 [1.05‐1.30]) increased the likelihood of choosing internal medicine. However, the intent to inherit an existing practice was associated with a lower likelihood of choosing internal medicine (OR 0.50 [0.31‐0.79]). While students of internal medicine had positive perceptions about terminal care (OR 1.14 [1.00‐1.30]) and clinical diagnostic reasoning (OR 1.50 [1.30‐1.73]), they were less interested in mastering advanced procedures (OR 0.79 [0.69‐0.92]) and acute care (OR 0.72 [0.63‐0.83]).

#### 3.3.2 General practice

In contrast to internal medicine, while general practice students were less likely to have a physician parent (OR 0.59 [0.42‐0.83]), the intent to inherit an existing practice was associated with general practice choice (OR 1.74 [1.06‐2.86]). Medical students who chose general practice preferred frequent patient communication (OR 1.21 [1.01‐1.46]), clinical diagnostic reasoning (OR 1.65 [1.40‐1.94]), and community‐oriented practice (OR 1.34 [1.14‐1.57]). Similar to internal medicine, general practice students rated the following variables as less important: mastering advanced procedures (OR 0.75 [0.64‐0.87]), acute care (OR 0.84 [0.73‐0.97]), and depth rather than breadth of practice (OR 0.68 [0.59‐0.80]).

#### 3.3.3 Pediatrics

Students who chose pediatrics showed interest in frequent patient communication (OR 1.38 [1.16‐1.64]). They responded that mastering advanced procedures (OR 0.76 [0.66‐0.88]) and depth rather than breadth of practice (OR 0.80 [0.69‐0.92]) were not as influential. Other demographic and career priority variables did not reach significance.

#### 3.3.4 Surgery

Female students were less likely to consider surgery as their specialty choice as compared to male students (OR 0.55 [0.39‐0.78]). Differences between surgery students and internal medicine/general practice students were largely driven by career priority variables in “primary care orientation” and “advanced and specific care.” For instance, surgery students were less interested in clinical diagnostic reasoning (OR 0.71 [0.60‐0.84]), which was one of the greatest drivers in choosing internal medicine and general practice. On the contrary, interest in mastering advanced procedures (OR 2.00 [1.66‐2.41]), acute care (OR 1.70 [1.44‐2.00]), and access to advanced medical fields (OR 1.56 [1.27‐1.90]) significantly increased the likelihood of choosing surgery. Surgery students were less likely to respond that work‐life balance was important (OR 0.70 [0.58‐0.83]) or that they had plans to open their own clinic (OR 0.77 [0.68‐0.88]).

#### 3.3.5 Emergency medicine

The strongest driver for choosing emergency medicine was a preference in acute care (OR 2.14 [1.79‐2.57]). In addition, students who chose emergency medicine shared several common features with surgery students. First, a significantly lower number of female students selected emergency medicine as their career option (OR 0.64 [0.45‐0.92]). Moreover, emergency medicine students responded that work‐life balance (OR 0.82 [0.69‐0.98]) and income (OR 0.83 [0.70‐0.99]) were less influential. On the other hand, interest in clinical diagnostic reasoning (OR 1.20 [1.01‐1.43]) and breadth of practice were similar to the general practice students, which was one of the characteristic differences between emergency medicine and surgery.

## 4 DISCUSSION

In 2020, a total of 9082 residents began residency training under the new board certification system in Japan. The number of new residents for each specialty in 2020 were as follows: internal medicine: 2923, surgery: 829, orthopedics: 671, pediatrics: 565, obstetrics and gynecology: 476, anesthesiology: 455, ophthalmology: 344, urology: 323, dermatology: 304, emergency medicine: 279, otolaryngology: 266, radiology: 247, neurosurgery: 247, general practice: 222, plastic surgery: 215, pathology: 102, rehabilitation: 83, and clinical examination: 14.^18^ According to a survey conducted by the Japanese Medical Specialty Board, approximately 85% of young physicians chose their specialty during medical school and junior residency.^19^ Thus, factors that elucidate the choice of medical specialty among medical students are of great significance, especially in recruitment for each specialty.

Previous studies have demonstrated several factors associated with medical students’ specialty choice. Kassebaum et al^3^ have demonstrated that medical school graduates who chose primary care tended to be female and older, had a rural background, and preferred intellectual challenges. Another study conducted at the University of Wisconsin revealed that primary care students placed emphasis on understanding populations, relationships with patients, and scope of practice, while salaries and competitiveness were not as important to them.^20^ Vaidya et al found that surgery, emergency medicine, and gynecology and obstetrics students demonstrated a higher “novelty‐seeking” tendency. They also reported that surgery students had lower “harm avoidance” and “reward dependency” scores. In contrast, students who chose primary care, emergency care, and gynecology and obstetrics had a high “reward dependency”.^8^

As aforementioned, the concept by Weiss et al^10^ that medical students may choose a cluster of related specialties based on a cluster of socioeconomic and occupational features might be particularly useful in understanding the process of specialty choice. This concept was supported by more recent study by Takeda et al^9^, which revealed several clusters of specialty based on the career preference clusters such as “fulfilling life with job security,” “bioscientific orientation,” and “personal reasons”. Our exploratory factor analysis and the subsequent multilevel logistic regression analyses revealed that career priorities under the “primary care orientation” category had positive association with choosing general practice, emergency medicine, internal medicine, and pediatrics, all of which could potentially have some primary care aspects. The “advanced and specific care” career priorities facilitated surgery and emergency medicine choices while reducing the likelihood of less procedure‐oriented specialties, such as internal medicine, general practice, and pediatrics. Intriguingly, those who had chosen “advanced and specific care”–related specialties (surgery and emergency medicine) rated lower perceived importance in “personal life orientation.”

Our results may imply that individualized career support based on student's preference in three simple factors, “primary care orientation,” “advanced and specific care,” and “personal life orientation,” in addition to knowing key differences among the cluster of related specialties, may be beneficial to facilitate the recruitment process in each specialty field.

This study has several limitations. First, the cross‐sectional data might not reflect medical students’ actual career choice. Thus, our models need to be validated in the future using longitudinal cohort of medical school graduates. Secondly, social desirability response bias may have led to ceiling effects on several career priority variables, which could undermine the discrimination capacity of the models. The relatively large sample size may have amplified the small effects. In addition, our results may not be applicable to junior residents since the study was limited to undergraduate medical education.

Our results demonstrated medical students’ career priorities and their association with specialty preference, using the concept of a cluster of related specialties and a cluster of career priority features. Since the majority of students have several career options, using the cluster of career priority features in addition to knowing key differences among the cluster of related specialties may be beneficial for both medical students and each specialty field, as well as for those considering interventions to manage the medical workforce.

## CONFLICT OF INTEREST

The authors declare no conflict of interests for this article.

## References

[1] The Japanese Ministry of Health L and W . Clarification of predicted number of physicians required in each specialty. Available from: https://www.mhlw.go.jp/content/10801000/000480275.pdf

[2] The Japanese Medical Specialty Board . About The Japanese Medical Specialty Board. Available from: https://www.japan‐senmon‐i.jp/

[3] Kassebaum DG , Szenas P , Schuchert MK . Determinants of the generalist career intentions of 1995 graduating medical students. Acad Med. 1996;71:198–209. 8615940 10.1097/00001888-199602000-00030

[4] Kassebaum PS . Factors influencing the specialty choices of 1993 Medical School Graduates. Acad Med. 1994;69(2):164–70. 10.1097/00001888-199402000-00027 8311892

[5] Kawamoto R , Ninomiya D , Kasai Y , Kusunoki T , Ohtsuka N , Kumagi T , et al. Gender difference in preference of specialty as a career choice among Japanese medical students. BMC Med Educ. 2016;16(1):1–8. 27829461 10.1186/s12909-016-0811-1 PMC5103608

[6] Kawamoto R , Ninomiya D , Kasai Y , Kusunoki T , Ohtsuka N , Kumagi T , et al. Factors associated with the choice of general medicine as a career among Japanese medical students. Med Educ Online. 2016;21:29448. 27172894 10.3402/meo.v21.29448 PMC4865794

[7] Ie K , Murata A , Tahara M , Komiyama M , Ichikawa S , Takemura YC , et al. What determines medical students’ career preference for general practice residency training?: a multicenter survey in Japan. Asia Pac Fam Med. 2018;17(2). 10.1186/s12930-018-0039-9 PMC5787259 29422773

[8] Vaidya NA , Sierles FS , Raida MD , Fakhoury FJ , Przybeck TR , Cloninger CR . Relationship between specialty choice and medical student temperament and character assessed with Cloninger inventory. Teach Learn Med. 2004;16(2):150–6. 15294460 10.1207/s15328015tlm1602_6

[9] Takeda Y , Morio K , Snell L , Otaki J , Takahashi M , Kai I . Characteristic profiles among students and junior doctors with specific career preferences. BMC Med Educ. 2013;13(1):1–11. 24028298 10.1186/1472-6920-13-125 PMC3847686

[10] Weiss YG , Zisk‐Rony RY , Tandeter H , Elchalal U , Avidan A , Schroeder JE , et al. Using medical specialty and selection criteria clusters to study specialty selection by Israeli medical students. BMC Med Educ. 2017;17(1):17. 28100274 10.1186/s12909-017-0854-y PMC5241925

[11] Wickham H , Miller E . Import and Export “SPSS”, “Stata” and “SAS” Files. [Internet]. R package version 2.1.0. 2019. [cited 2019 Aug 5]. Available from https://cran.r‐project.org/package=haven

[12] Wickham H . Easily Install and Load the “Tidyverse”. R package version 1.2.1. 2017.

[13] Revelle W .Procedures for Personality and Psychological Research. R package version 1.8.12. 2018.

[14] Bernaards CA , Jennrich R . Gradient projection algorithms and software for arbitrary rotation criteria in factor analysis. Educ Psychol Meas. 2005;65:676–96.

[15] Bates D , Maechler M , Bolker B , Walker S . Fitting linear mixed‐effects models using lme4. J Stat Softw. 2015;65:1–48.

[16] Robin X , Turck N , Hainard A , Tiberti N , Lisacek F , Sanchez JC , et al. pROC: an open‐source package for R and S+ to analyze and compare ROC curves. BMC Bioinform. 2011;12:77. 10.1186/1471-2105-12-77 PMC3068975 21414208

[17] Yoshida K . tableone: Create “Table 1” to Describe Baseline Characteristics. R package version 0.10.0. 2019.

[18] The Japanese Ministry of Health L and W . Comparison of the number of residents in 2018 [Internet]. 2019 [cited 2019 Aug 17]. Available from https://www.mhlw.go.jp/content/10803000/000452411.pdf

[19] Japan Medical Association Research Institute . Survey on career intention of medical students [Internet]. 2015 Available from http://www.jmari.med.or.jp/download/WP337.pdf

[20] Knox KE , Getzin A , Bergum A , McBride P , Rieselbach R , Friedsam D . Short report: factors that affect specialty choice and career plans of {Wisconsin}’s medical students. WMJ Off Publ State Med Soc Wisconsin. 2008;107(8):369–73. 19331006

